# Acceptability and Reliability of the Bayley Scales of Infant and Toddler Development-III Among Children in Bhaktapur, Nepal

**DOI:** 10.3389/fpsyg.2018.01265

**Published:** 2018-07-24

**Authors:** Suman Ranjitkar, Ingrid Kvestad, Tor A. Strand, Manjeswori Ulak, Merina Shrestha, Ram K. Chandyo, Laxman Shrestha, Mari Hysing

**Affiliations:** ^1^Child Health Research Project, Department of Pediatrics, Tribhuvan University Teaching Hospital, Kathmandu, Nepal; ^2^Regional Centre for Child and Youth Mental Health and Child Welfare, Uni Research Health, Bergen, Norway; ^3^Department of Research, Innlandet Hospital Trust, Lillehammer, Norway; ^4^Centre for International Health, University of Bergen, Bergen, Norway; ^5^Department of Community Medicine, Kathmandu Medical College, Kathmandu, Nepal

**Keywords:** Bayley scales of infant and toddler development-III, reliability, psychometric properties, LMIC, neurodevelopment

## Abstract

**Background:** The Bayley Scales of Infant and Toddler Development, 3rd edition (Bayley-III) is the most widely used developmental assessment tool for infants and toddlers worldwide, but less is known about its psychometric properties and feasibility in low and middle-income countries.

**Aim:** To assess the psychometric properties and feasibility of the Bayley-III when used in a large scale randomized controlled intervention trial in Nepal.

**Methods:** The participating infants were part of a randomized, doubled blind, placebo-controlled trial to measure the efficacy of vitamin B12 supplementation on growth and neurodevelopment. A total of 600 children aged 6–11 months were enrolled and included for developmental assessment. The Bayley-III measures child development across five domains: cognition, receptive and expressive language, fine and gross motor skills. Some items were culturally adapted. To measure and ensure appropriate inter-observer agreement, standardization exercises were performed during the initial training, and double scoring of 7% of test sessions were conducted throughout the study by two examiners.

**Results:** The inter-rater agreement was excellent for both the standardization exercises before the start of the study, and for the quality control throughout the study with intraclass correlation coefficient ranging from 0.95 to 0.99. The internal consistency measured by the Cronbach’s alpha coefficient ranged between 0.57 and 0.87. The subtests raw scores as well as scaled scores were significantly correlated (*p* < 0.001). The means and SDs of the scaled scores compared with American norms were similar to the distribution in the American sample, with the exception of the receptive (Mean = 7.7, SD = 2.2) and expressive (Mean = 7.3, SD = 1.9) language subtests that were lower than the American norms.

**Conclusion:** The inter-rater reliability between the scorers on the Bayley-III was excellent both during standardization and for the quality control. The distributions for the cognitive and motor subscales are comparable to the American norms, while caution is needed in the interpretation of the language scales. The results suggest that Bayley-III is a feasible tool for the assessment of neurodevelopmental status in nutritional studies in low resource settings such as Nepal. Cultural adaptations, training and standardization are prerequisites for a valid and reliable assessment using the Bayley-III.

## Introduction

The Bayley Scales of Infant and Toddler Development (Bayley) is a widely used developmental assessment tool, and often considered the gold standard in the assessment of early child development (Walder et al., 2009). The Bayley scales have its origin in the United States and have norms based on an American sample (Bayley, 2006a), but are used worldwide to assess child development in both clinical practice and research studies (Albers and Grieve, 2007; Torras-Mañá et al., 2016; Ballot et al., 2017). However, the Bayley scales and other measures developed in Western high-income countries, may not be as valid and reliable when used in eastern societies and low and middle-income countries (LMIC). The psychometric properties in these settings need further investigation (Sternberg and Grigorenko, 2004).

Cultural adaptations are often needed when using developmental assessment tools constructed in western societies in new cultural settings. Some of the objects used in developmental assessments, as for instance, stairs and scissors may not be familiar in some cultures. The use of American norms for children from LMIC may also be problematic. Previous studies have found that there are differences in developmental scores across countries and cultures when comparing results with the United States norms (Murray-Kolb et al., 2014; Steenis et al., 2015). For instance, gross motor function was comparably better in Cameroonian children, while German children had more advanced language development (Lohaus et al., 2011). Nepalese children had lower performance in cognitive and motor development compared to the United States norms in a study from rural Nepal (Manandhar et al., 2016). Finally, appropriate training and standardization is a prerequisite to obtain reliable assessment across testers.

The main aim of the present study was to assess the psychometric properties of the Bayley 3rd edition (Bayley-III) when used in the field study setting of Nepal, including the feasibility of the test, the reliability of assessments across tester, and to compare the results to the American norms.

## Materials and Methods

### Participants

The sample was part of a randomized, double-blinded, placebo-controlled trial to measure the efficacy of routine administration of vitamin B12 on growth and neurodevelopment (Strand et al., 2017). A total of 600 children were enrolled, and developmental outcomes of each child were assessed at baseline. For the enrollment to the main study, a door-to-door survey was conducted to identify households with children aged 6–11 months. Children were screened for eligibility by a physician and field supervisors. Children were enrolled after obtaining informed consent.

### Study Design, Setting, and Population

This is a superiority, parallel group, community-based, individually randomized, double-blinded, placebo-controlled trial. ClinicalTrials.gov Identifier: NCT02272842.

The study site is the urban and surrounding communities of Bhaktapur municipality in Nepal. Inclusion criteria were (1) length for age *z*-score < -1, (2) plan to reside in the Bhaktapur municipality and surrounding areas in the district for the next 12 months, and (3) availability of informed consent.

Exclusion criteria for the children were (1) severe systemic illness requiring hospitalization, (2) severe malnutrition (weight for length < -3 *z*-scores), (3) taking supplements that include vitamin B12, (4) severe anemia (Hb < 7 g/dL), and (5) ongoing acute infections such as fever that required medical treatment. The last two were temporary exclusion criteria and the children could be enrolled after recovery. For more information on the study, see the protocol paper (Strand et al., 2017).

### Instrument

The Bayley-III is an individually administered assessment tool of global developmental status for children aged 1–42 months (Bayley, 2006a). The test takes 45–60 min to administer, and consists of three domains: the Cognitive, Language (receptive and expressive communication), and Motor (fine and gross motor) domain. Each test item is scored credit or no credit according to the manual, and the credited scores are summed for the total raw scores for each scale. The ceiling rule of this test is that the test continues until five consecutive scores of no credit.

The Bayley raw scores are converted into scaled scores based on the American norms (Bayley, 2006b). The American norms are from a representative American sample based on stratification on parental education, proportions of Whites, African Americans, Hispanics, Asians, and other racial/ethnic groups, and geographic regions of the United States. The standard scaled scores have a mean of 10, a SD of 3, and a range from 1 to 19. Thus, based on the American norms, a subtest scaled score of 10 reflects the average performance of a given age group in the American sample.

#### Translation and Cultural Adaptation

The Bayley-III for the current study was initially adopted for the Malnutrition and Enteric Diseases (Mal-ED) study in the same population in children from 6 to 24 months (Murray-Kolb et al., 2014). Test instructions, some items of the language subtests and the social-emotional subtests (not included in this paper) were translated and back-translated according to standard procedure. The test materials were reviewed and adaptations were done to assure cultural relevance and then piloted in the population.

The Initial translation was done by a team of psychologists and pediatricians familiar with the local language and culture, and with more than 10 years of experience in research and clinical practice. The back-translation was carried out by a person fluent in English language who was not connected to the team that conducted the original translation.

For the adaptation, testing materials were reviewed in terms of cultural relevance, and eventually substitutions were identified, discussed, and agreed on in a larger study team with experts from the MAL-ED study. Adapted items were piloted on approximately 20–30 children prior to the start of formal testing.

Adaptations were applied to some of the items in the language domain by matching the style of the original item by identifying cultural appropriate photographs and cartoons when needed. For example, a photograph of a person vacuuming was replaced by a photograph of a woman using a broom since vacuum cleaners are not common in the study area (Murray-Kolb et al., 2014).

We also did some modification to the test materials. The Bayley Scales include a “picture book” with photographs of objects and actions. As the photographs mostly depicted objects in a Western setting, a version more relevant for a Nepalese setting was developed. For example, a mother giving an oil massage to her baby was used instead of a woman swimming with her baby in a pool, and a woman washing clothes in a bucket was used instead of a man using the washing machine. Similarly, an adapted version of the Bayley stimulus book was developed by an artist. Some of the drawings of objects and actions were modified to be relevant to a Nepalese setting and to depict Nepalese people. For instance, a boy and a girl bathing in a tap instead of swimming in a pool, and a child making snowman was changed to a child making a toy with clay. Other pictures were also adapted with pictures that were similar to a Nepalese setting.

#### Training and Quality Control

A local psychologist with 4 years’ experience in Bayley-III assessments for research, served as the gold standard both during training and throughout the study. This psychologist was also responsible for training of the other psychologists for the study. A neuropsychologist and a clinical child psychologist from Norway supervised the training and standardization. During the 5 days of training, administration and understanding of the inherent ideas of items and scoring, as well as approaches and techniques in terms of rapport building were discussed and practiced.

Before enrollment, standardization exercises were performed in 20 children where the Bayley assessments were scored in doubles. The raters were required to reach a high inter-rater reliability (ICC > 0.90) before starting to perform the assessments. About 7% of the sessions during the main study were also double scored in real-time by the gold standard to measure and ensure appropriate inter-observer agreement. In addition to this, all the Bayley-III assessments were recorded on video. These videos were primarily used for support in scoring but were also used for additional checks by the supervising psychologists for prompt feedback to the assessors. During the study period, weekly Skype-meetings were held with the team to discuss the progress in general and challenges that were being faced; particular issues with the Bayley-III testing where also be discussed in these meetings.

#### Test Situation

Children were tested in the presence of their mothers, or another caregiver. Ahead of testing, we ensured that the children were well fed and not sick. The testing was done at the field office in a room that is well-lit, well-ventilated, and free from any distractions according to the standard of the testing of Bayley. Cognitive, receptive, and expressive language and fine motor subtests were assessed with the children on mother’s or caregiver’s lap sitting in front of the psychologist by a table, while the gross motor subtest was assessed on the floor.

The examiner started with rapport building with the child while the mother or caregiver was instructed about the assessment. The test was then administered according to the manual of the Bayley-III. Breaks were given during the assessment for feeding, rest, and/or nap when needed. The number of breaks were not fixed and varied according to the child’s need. Video recordings were carried out by a single stationary camera which can cover all the assessment. Specially, cognitive, language, and fine motor subtests were carried out on the table sitting on the mother’s lap in front of the assessor. For gross motor, the camera angle was adjusted to capture all the activities of the children (sitting, crawling, standing, walking, etc.).

### Statistical Analysis

Demographic characteristics of the children were initially summarized using means and SDs if generating continuous data, and using frequency counts and percentages if generating categorical data.

Inter-rater reliability between the two psychologists for the standardization sample and for the quality control was expressed using the intraclass correlation coefficient (ICC). Additionally, correlations between the raw scores and scaled scores of the different subtests were expressed by Pearson’s product-moment correlations coefficients. Internal consistency of the test was expressed as the Cronbach’s alpha for itemized data. The 95% CIs of the Cronbach’s alpha and correlation coefficients were calculated using bootstrap resampling. The alpha values from < 0.6, 0.6 to 0.8, and > 0.8 were set as questionable, acceptable, and good, respectively. Furthermore, means and SDs of the scaled scores were calculated for the subscales of the Bayley-III, which were then compared with the American norms. All the subtest scaled scores were further analyzed individually based on age of children in months and graphs were obtained for each subtest. Data was analyzed using the STATA 15.0 software (STATA, College Station, TX, United States).

## Results

### Demographic Characteristics

The demographic characteristics of the participants are shown in **Table 1**. A total number of 600 children were enrolled. The mean (SD) age at enrollment was 8 (1.7) months and about 48% were female. About 10% of the children were born preterm (before 37 weeks of gestational period). The mean (SD) birth weight of the children was 2787 gram (497), and about 19% were born with birth weight less than 2500. Exclusively breastfed up to 6 months of age were about 10%. The sample comprised mostly of children from the following ethnic groups: Newars (70%), Tamangs (16%), and Brahmin/Chhetri (8%). About 35% of both mothers and fathers had an education level up to grade 5. Most of the mothers (62%) were house-wives and only about 5% of the fathers were not employed. The ratio of the family type is almost equal, 51% from nuclear families and 49% from joint families.

**Table 1:** Demographic characteristics of participants (*N* = 600).

Child	
Mean age of child in months (SD)	8 (1.7)
Female child (%)	291 (48.5)
Gestational week	
Preterm (< 37 weeks of gestation)	62 (10.4)
Full term	523 (87.17)
Post term	14 (2.33)
Birth order (%)	
1st	292 (48.7)
2nd	229 (38.1)
3rd	79 (13.2)
Mean birth weight in grams (SD)	2787 (497)
Exclusive breastfeeding for 6 months or more	64 (10.6)
Family type	
Nuclear	308 (51.3)
Joint	292 (48.7)
**Mother**	
Mean age (SD)	27 (4)
Literacy	
Illiterate up to grade 5	211 (35.5)
Grade 5–10	130 (21.8)
Grade 11 and 12	148 (24.8)
Bachelor	73 (12.2)
Master or above	33 (5.5)
Occupation	
No working mother/Agriculture	371 (61.8)
Carpet worker	17 (2.8)
Daily wage earner	73 (12.2)
Services	73 (12.2)
**Father**	
Mean age (SD)	25 (8)
Literacy	
Illiterate up to grade 5	221 (37)
Grade 5–10	113 (19)
SLC to grade 12	148 (24.8)
Bachelor	88 (14.7)
Master or above	26 (4.3)
Occupation	
No working/Agriculture	32 (5.3)
Carpet worker	7 (1.2)
Daily wage earner	233 (39)
Services	113 (19)
Self employed	173 (29)


### Reliability and Validity

The inter-rater reliability coefficients were excellent for both the standardization sample (ICC = 0.99) and for the quality control throughout the study (ICC = 0.95–0.99) (**Table 2**). For the internal consistency measures, the Cronbach’s alphas ranged between 0.57 and 0.87 (**Table 3**). The cognitive subtest and gross motor subtest showed good reliability while the receptive and fine motor had alpha values indicating acceptable internal consistency. The expressive communication subtest had the lowest alpha value of the different scales.

**Table 2:** Inter-rater reliability between the two psychologists on the Bayley-III subtests for the standardization sample and for the quality control.

Bayley	Standardization (*N* = 30), Non-study children	Quality Control (*N* = 41), study children

Subtest	ICC	ICC (CI)
Cognitive	0.99 (0.99, 1.00)	0.99 (0.99, 1.00),
Receptive Language	0.99 (0.99, 1.00)	0.97 (0.96, 0.99)
Expressive Language	0.99 (0.99, 1.00)	0.98 (0.97, 0.99)
Fine Motor	1.00 (1.00, 1.00)	0.99 (0.98, 0.99)
Gross Motor	0.99 (0.99, 1.00)	1.00 (0.99, 1.00)


**Table 3:** Cronbach’s Alphas (95% CI) for the Bayley-III items in each subtest.

Bayley Subtest	Mean Alpha coefficient
Cognitive	0.84 (0.46–1.22)
Receptive Language	0.57 (-0.14–1.28)
Expressive Language	0.66 (0.29–1.03)
Fine Motor	0.72 (0.55–0.89)
Gross Motor	0.87 (0.79–0.96)


*α > 0.8, Good; 0.7 ≤ α < 0.8, Acceptable; 0.6 ≤ α < 0.7, Questionable; and 0.5 ≤ α < 0.6, Poor. Cronbach’s alphas were calculated using the alpha command in STATA. The 95% CI were estimated using bootstrap resampling.*

The correlations between the subtests raw scores are presented in **Table 4**, and the scaled scores in **Table 5**. The cognitive subtest showed significantly strong correlation with fine motor and gross motor subtests. Similarly, fine and gross motor subtests were strongly correlated and the rest of the subtests showed moderate correlations. With scaled scores, all the subtests showed significantly weak to moderate correlations. **Table 6** show the means (SD) raw scores for each subtest according to age.

**Table 4:** Inter-correlations of the raw scores in each subtest.

	Cognitive	Receptive	Expressive	Fine Motor	Gross Motor
Cognitive	1				
Receptive	0.47 (0.41, 0.54)	1			
Expressive	0.63 (0.58, 0.67)	0.47 (0.39, 0.54)	1		
Fine Motor	0.81 (0.78, 0.83)	0.45 (0.39, 0.52)	0.56 (0.51, 0.62)	1	
Gross Motor	0.78 (0.75, 0.81)	0.45 (0.38, 0.52)	0.60 (0.56, 0.65)	0.76 (0.73, 0.79)	1


*Pearson’s correlation coefficients. The 95% CI were estimated using bootstrap resampling.*

**Table 5:** Inter-correlations of the scaled scores in each subtest.

	Cognitive	Receptive	Expressive	Fine Motor	Gross Motor
Cognitive	1				
Receptive	0.21 (0.13, 0.29)	1			
Expressive	0.23 (0.15, 0.30)	0.24 (0.16, 0.32)	1		
Fine Motor	0.50 (0.42, 0.57)	0.24 (0.16, 0.31)	0.14 (0.06, 0.21)	1	
Gross Motor	0.35 (0.28, 0.42)	0.33 (0.26, 0.40)	0.21 (0.13, 0.29)	0.39 (0.32, 0.45)	1


*Pearson’s correlation coefficients. The 95% CI were estimated using bootstrap resampling.*

**Table 6:** Participants mean (SD) of raw scores in the 600 Nepalese infants.

Month		Cognitive		Receptive		Expressive		Fine Motor		Gross Motor	
						
	*N*	Mean	SD	Mean	SD	Mean	SD	Mean	SD	Mean	SD
6	175										
		26.86	2.89	9.25	1.14	6.2	1.16	19.23	2.70	24.83	3.37
7	161										
		29.91	2.27	9.33	1.11	6.69	1.4	21.78	1.96	27.91	3.18
8	84										
		31.5	2.89	9.85	1.25	7.49	1.88	22.85	1.87	30.1	4.26
9	86										
		34.16	2.76	10.18	1.2	8.47	2.03	24.43	1.95	33.11	2.91
10	84										
		35.88	2.69	10.29	1.25	9.37	2.18	25.32	1.95	34.66	3.37
11	86										
		38.29	3.03	10.96	1.63	10.63	2.53	27.34	2.49	37.94	3.96


### Comparisons With United States Norms

**Table 7** describes the mean scaled scores for the cognitive, receptive and expressive language, and the fine and gross motor subtests. All mean scores and CIs are below 10 with a range between 7.29 and 9.55 and *p*-values for all comparisons are > 0.001. The effect sizes between the American norms and the observed Nepali scores ranged from small effects for the cognitive and the fine motor subtest, medium for the gross motor, and large effect sizes for both language subtests.

**Table 7:** Mean (SD) scaled scores and Cohen’s D for the Bayley-III subtests based on the American norms (*N* = 600).

Subtests	*N*	Mean	*SD*	Range	CI		Cohen’s D
Cognitive	600	9.55	2.05	2–16	9.38	9.71	0.18
Receptive	600	7.67	2.17	1–14	7.49	7.84	0.89
Expressive	600	7.29	1.90	1–14	7.14	7.44	1.08
Fine Motor	600	9.49	2.34	1–17	9.30	9.68	0.19
Gross Motor	600	8.86	2.64	1–17	8.65	9.07	0.40


There are significant lower scaled scores for children with low birthweight compared to normal birthweight, and preterm birth compared to term birth, except for the language subtests (**Table 8**).

**Table 8:** Mean (SD) scaled scores for the Bayley-III subtests according to birth weight and gestational age based on the American norms.

Subtests	*N*	Mean score ≥ 2500 gm	SD	*N*	Mean score < 2500 gm	SD	*p*-value	*N*	Mean score > 37 weeks	SD	*N*	Mean score < 37 weeks	SD	*p*-value
Cognitive	464	9.82	1.85	115	8.4	2.84	<0.001	538	9.68	1.97	62	8.39	2.36	<0.001
Receptive	464	7.69	2.11	115	7.6	2.36	0.429	538	7.64	2.17	62	7.87	2.15	0.677
Expressive	464	7.36	1.92	115	7.01	1.80	0.139	538	7.33	1.91	62	6.95	1.83	0.086
Fine Motor	464	9.69	2.17	115	8.61	2.78	0.003	538	9.61	2.24	62	8.47	2.87	<0.001
Gross Motor	464	9.08	2.54	115	8.04	2.74	0.004	538	8.96	2.61	62	7.97	2.69	<0.001


**FIGURE 1:**
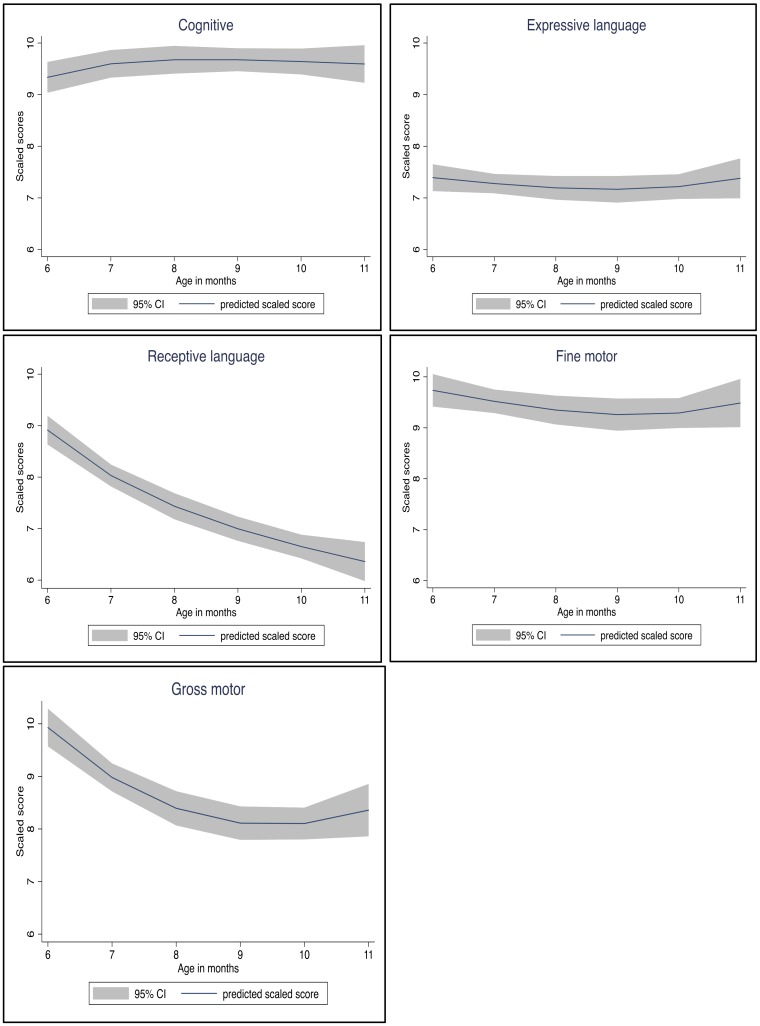
Subtests scaled scores by age in months at testing.

**Figure 1** demonstrates how the mean scaled scores for the different subtests vary by age. In the receptive language, the mean scaled score lowered from 9.00 at 6 months of age to 6.28 at 10 months, and slightly increased to 6.44 at 11 months. A similar trend was seen in the gross motor subtest, where the mean scaled score lowered from 9.91 at 6 months of age to 7.76 at 10 months and increased to 8.44 at 11 months. For the other subtests, the mean scaled scores showed a more consistent trend at the different ages.

## Discussion

In the present study, we assessed the psychometric qualities of the Bayley-III in an adapted version to a Nepalese study setting. The distributions for the cognitive and motor subtests scaled scores are comparable to the American norms, while caution is needed in the interpretation of the scaled scores on the language subtests. The inter-rater reliability was excellent between the scorers both during standardization and for the quality control throughout the study. A study in rural Nepal has also confirmed high inter-rater reliability for the motor and cognitive domains, while language scales were not assessed (Manandhar et al., 2016). Overall the results suggest that the Bayley-III can be a feasible tool for the assessment of neurodevelopmental status in Nepalese children. Similar results have been shown in a study of preterm infants in Taiwan that demonstrated good to excellent inter-rater reliability (Yu et al., 2013), and good inter-rater reliability in a South African cohort study (Ballot et al., 2017). Taken together, this supports that the Bayley-III can be used reliably with multiple raters in large studies. However, competent testers, as well as a thorough training, standardization and quality control are prerequisites for these high inter-rater correlations, which are characteristics of all the aforementioned studies.

As shown by the assessment of internal consistency by the Cronbach’s alpha, the reliability of the scores on the cognitive subtest was good, the fine motor subtests acceptable and gross motor good, while the receptive and expressive language scales showed poor and questionable internal consistency. Other studies that included Ethiopian, Malaysian, and Persian speaking children also found similar results with the reliability ranging from questionable to good (Zakaria et al., 2012; Hanlon et al., 2016; Azari et al., 2017). Thus, the assessment of reliability at the subtest level should be undertaken in cross-cultural studies to ensure reliable measurements. For both clinical and research purposes, the present study suggest that the language scales need to be interpreted with caution due to the low alpha values questioning the reliability of these subscales. This was further confirmed by the lack of differences between the high and low risk groups on the language scales.

The construct validity of the Bayley was supported through the strong correlation between the subtests based on raw scores and low to moderate for the scaled scores. Our results are similar to the results in a Malay version of the Bayley reliability and validity study, showing low to moderate correlations between the subtests (Zakaria et al., 2012).

The mean levels of the subtest scaled scores were all lower than the American norms, with small effect sizes for the cognitive and fine motor, medium for the gross motor subtest, while the language scales had differences with large effect sizes. The results are in line with previous studies that find significant differences between scores of Dutch children and the American norms (Steenis et al., 2015), Malawian children in comparison to the United States norms (Cromwell et al., 2014), and Taiwanese preterm children (Yu et al., 2013). A range of factors may have affected the discrepancy between the mean scaled scores in the present study from the United States norms. Lack of appropriate cultural adaptations may be one important factor leading to this discrepancy, which was demonstrated in a study in Malawian children where the comparison of the scores with the United States norms prior to cultural adaptation resulted in misclassification of developmental delay (Cromwell et al., 2014).

Children scored very low in both receptive and expressive language as compared to other domains, and this difference increased over time. The language scales also showed lower reliability. Taken together, caution should be taken when using the Bayley-III language scales for Nepali children in this age group. The reasons for the limitations on the language subtest could be related to many factors. The cultural adaption and translation is one possibility since the language subscales are the hardest to properly adapt. For instance, items in the language subtest relating to “Understands pronoun” are difficult to administer in our study setting since both in Nepali and Newari language there is a lack of proper words for the pronouns “His” and “Her.” Many children are bilingual which is also known to be related to language development (Hoff and Ribot, 2017). Further, the lack of vocalization in the test setting may also have impacted the results. The low rate of vocalization was also experienced in an Indian study using the Ages and Stages Questionnaire 3rd edition where most of the language items relied on caregiver’s response because the children did not vocalize at the time of testing (Kvestad et al., 2013). The lack of vocalization may be related to lack of knowledge in the mothers. In a study from the same study, population result shows that most of the Nepalese mothers responded that they should start talking with children at the age of 11.5 ± 8.6 months, and very few mothers knew when to start shared book reading (Shrestha et al., 2017). A study conducted in South Africa has also shown similar results; in comparison with the baseline and 1 year after follow-up Bayley, the Bayley language score decreased from baseline to 1 year follow-up probably because the children were not exposed to books (Ballot et al., 2017).

The present study includes a sample of children with high-risk of developmental delay such as marginally stunted children (Mendez and Adair, 1999; Walker et al., 2005; Crookston et al., 2010; Scharf et al., 2015; Sudfeld et al., 2015) and with a high rate of preterm children. These high risk groups could account for the relatively low developmental level compared to the United States norms (Yu et al., 2013; Beauregard et al., 2018). Except for in the language subtests, premature children had lower scores than the full-term children (Menyuk et al., 1991; Cattani et al., 2010). Similarly, the children born with low birth weight also showed significantly lower scores, providing support to that low birth weight could affect the development in early life (Tong et al., 2006).

Parent-related factors may also influence the test performance. Parental responsiveness and support for children’s development is related to cognitive development (Landry et al., 2001; Tamis-LeMonda and Rodriguez, 2008; Gauvain et al., 2011), and thus, the low score of children in all domains compared to the United States norms could also be due to child rearing practices in Nepalese context. In a previous study in the same study setting, most of the mothers showed lack of knowledge on the appropriate timing to provide different stimulation activities (Shrestha et al., 2017). Thus, children may have received less opportunities to explore new things such as toys at a young age compared to children in the American normative sample.

About 35% parents in the present study were illiterate/or had an educational level up to grade 5. As a consequence, the low scores in the present study might also be because of parental education, knowing that there is a social gradient in child development, with lower cognitive levels at lower levels of socioeconomic status (Hart and Risley, 1995; Bradley and Corwyn, 2002; Tamis-LeMonda and Rodriguez, 2008; Letourneau et al., 2013; Christensen et al., 2014; Von Stumm and Plomin, 2015; Playford et al., 2017).

Finally, our sample covers 70% of Newar children that are known to be lower on modernity than other ethnic groups. The mean scores lower than the United States norms could thus be attributable to their less exposure to modernity, predicted by various facilities of resources, technology, and communication with the world outside the community. This is supported by a study that found that larger modernity ratings in a household, showed relatively higher scores in cognitive development (Gauvain and Munroe, 2009).

### Strengths

The large sample of 600 children is one of the main strengths of this study. Further, the cultural adaption of the items, and that the tool has been used in a previous developmental study in the same population (Murray-Kolb et al., 2014) represents strengths of the study. Before starting the assessments, standardization practices were done with gold standard to ensure quality of the data. During the study period, double scorings were performed with the gold standard to maintain the quality of the data and prevent examiners drift.

### Limitation

The sample is a high-risk sample that is part of a clinical trial, and thus, it is not a population-based sample, and care should be taken before generalizing to the population as a whole.

## Conclusion

The inter-rater reliability was excellent between the scorers on the Bayley-III both during standardization and for the quality control. The internal consistency between all the subtests is moderate to high, and the subtests showed low to moderate correlation with each other. The distributions for the cognitive and motor subtests are comparable to the American norms, while caution is needed in the interpretation of the language subtests.

The results suggest that Bayley-III can be a feasible instrument for developmental assessment for Nepalese children between 6 months and 11 months. Cultural adaptations, training, and standardization are prerequisites for a valid and reliable assessment using the Bayley-III.

## Ethics Statement

Ethics clearances have been obtained from the National Health and Research Council (NHRC; No. 233/2014) in Nepal and from the Regional Committee for Medical and Health Research Ethics (REC; No. 2014/ 1528) in Norway. The parents/guardians of infants eligible for the trial are asked for written informed consent or for a thumbprint (in the presence of an impartial witness) if they are illiterate, declaring their willingness to have their infant participate in the trial. The participant information sheet is in the local language and describes in detail the focus of the study along with the associated risks and benefits for the infant.

## Author Contributions

TS, MH, IK, and RC designed the study. RC, MU, SR, LS, and MS conducted the research and were responsible for the field implementation and data collection. TS, MH, and SR analyzed the data and interpreted the results. SR and MH had primary responsibility for the final content. All the authors read and approved the final manuscript.

## Conflict of Interest Statement

The authors declare that the research was conducted in the absence of any commercial or financial relationships that could be construed as a potential conflict of interest.
